# A pivot joint steering mechanism for tip-everting soft growing robots

**DOI:** 10.3389/frobt.2025.1627116

**Published:** 2025-07-17

**Authors:** Tianchen Ji, Zheyuan Bi, Lin Cao

**Affiliations:** School of Electrical and Electronic Engineering, The University of Sheffield, Sheffield, United Kingdom

**Keywords:** soft growing robot, actuation mechanism design, tendon actuated, confined space navigation, soft robot

## Abstract

Soft growing robots (SGRs) navigate confined environments by everting material from the tip while keeping the rest of the body stationary, enabling frictionless navigation. This opens up huge potential for inspection, search, and rescue tasks. However, controlling the direction of tip growth is still a challenge because of the ever-changing tip of the robot during tip growth. This study presents a compact steering mechanism that integrates a tendon-driven pivot joint with a pressure-tunable internal bladder. By modulating friction between the pivot joint and the inner material, the mechanism switches between two states: decoupled (stationary for bending) and coupled (move forward together with robot’s inner material). This enables the robot to bend locally and then continue growing in the new direction, without using complex full-body actuation or external mechanisms. A robotic platform was developed to implement this mechanism, and its performance was characterized and validated through modeling and experiments. Experimental results confirm that the mechanism achieves reliable tip steering, closely matches kinematics models, and interacts gently with the environment. The proposed design offers a scalable and structurally simple solution for long-range navigation in soft growing robots.

## 1 Introduction

Tip-everting Soft Growing Robots (SGRs), which can lengthen their bodies by everting their inner material at the tip (Hawkes et al., 2017), have emerged in recent years. Tip growth is a common phenomenon in plant growth, from pollen tubes (Steer and Steer, 1989) and plant roots to creeping vines (Weigel and Jürgens, 2002). This particular attribute of mobile growth allows the tip to navigate frictionlessly in a narrow and tightly restricted environment and to build a conduit with the aid of the surrounding environment (Sanati Nezhad and Geitmann, 2013). Without relative sliding with the environment, there is no friction between the robot and the environment, reducing the impact of the robot to the environment and enabling the robot to travel further (Blumenschein et al., 2020). This leads to broad application of SGR for minimally invasive surgery (Taylor and Stoianovici, 2003), small-environment inspection (Liu and Kleiner, 2013), rescue (Siciliano and Khatib, 2016), and archaeology (Coad et al., 2020).

For tip-everting SGRs, achieving reversible, localized steering during continuous eversion remains a core challenge. A steering unit must (i) lock to the body long enough to generate curvature, then (ii) decouple so new material can emerge. After bending, the shape must be held while fresh membrane everts, yet practical locking or environment-bracing solutions remain scarce. Moreover, few robots can shift the steering site downstream as they elongate without external insertion tools. Many approaches have been proposed to actively steer the Tip Everting SGR, such as (1) integrated pneumatic latch mechanism (Hawkes et al., 2017), (2) SPAM (Series Pneumatic Artificial Muscles) (Coad et al., 2020), (3) helical tendon routing (Blumenschein et al., 2018), (4) variable stiffness (Do et al., 2023), (5) Distributed Pneumatic Segment (der Maur et al., 2021), (6) Internal-Skeleton-Based Steering (Haggerty et al., 2021; Takahashi et al., 2021; Li et al., 2021; Qin et al., 2025), (7) Non-actuator-based strategies Mendoza et al. (2024), Davy et al. (2024).

Many approaches have been proposed to actively steer the Tip Everting SGR. The integrated pneumatic latch mechanism in SGR features a pneumatic chamber on each side with prelocked wrinkles controlled by a series of latches (Hawkes et al., 2017). Actuating a chamber releases a latch at the tip of the robot, creating different lengths on the sides of the SGR and allowing directional steering. This design ensures that the shape of the SGR remains constant during navigation, thus reducing the environmental impact. However, steering occurs only during growth and is irreversible; that is, once a direction is set, it cannot be changed.

The more commonly used SPAM (Series Pneumatic Artificial Muscles) approach involves a pneumatic chamber in the SGR body that contracts when pressurised, causing reversible bending (Coad et al., 2020). Unlike pneumatic latching mechanisms, SPAM allows for steering without growth and is reversible. However, it bends the entire SGR body, which is not always desirable, particularly when only tip steering is preferred. In constrained environments, SPAM-steered SGR may produce unpredictable steering owing to their interactions with their surroundings. In addition, the large size of pneumatic pouches makes the robots bulky and difficult to miniaturise, limiting their use in medical applications.

The helical tendon routing approach uses a tendon routed along the helical path around an inflated beam through a series of short tendon-guiding segments (Blumenschein et al., 2018). When pulled, the tendon reconfigures the inflated beam into a helical shape. This can only generate a structure of the desired shape, but is limited in providing arbitrary steering during navigation.

The variable stiffness approach uses layer jamming to dynamically change the stiffness of sections along an inflated continuum beam to selectively “activate” discrete joints (Do et al., 2023). By changing the activated joints, the output of a single actuator can be reconfigured to actively control the bending angles of various joints. This approach can remarkably change the shape of the SGR in many different sections; however, it requires many valves and pneumatic lines to separately control the layer jamming of each section, leading to a complex and bulky robot body design.

Another commonly adopted method involves distributed pneumatic segment actuation, in which multiple internal pneumatic chambers are embedded along the robot body. der Maur et al. (2021) developed RoBoa, a steerable vine robot driven by such distributed actuators. While this design enables effective steering and navigation, especially in search-and-rescue scenarios, it introduces mechanical complexity due to the need for pneumatic routing and synchronized control.

Internal-skeleton steering typically fixes a mechanical linkage to the inner membrane at two points, the two-contact position draw together as the actuation mechanism driven, forming a wrinkle that creates a length mismatch between the robot’s flanks and thus a bend. For single steering structure, Haggerty et al. (2021) drive the linkage with on-board electric motors, increasing mass and restricting use in environments sensitive to electronics; moreover, their tip-mounted storage scheme limits the availability of a central tool channel. Takahashi et al. (2021) require manual repositioning of the linkage relative to the base, which precludes fully autonomous operation. Li et al. (2021), Qin et al. (2025) and Wang et al. (2020) employ distributed embedded actuation network for steering and shape retention. Although highly dexterous, these electro-magnetic or pneumatic lattices increase design complexity and confine the effective growth length to the region that contains actuators, leaving unactuated segments unable to steer. Wang et al. (2020) introduced a variable-length shape-locking backbone that allows segment-wise bending by mechanically constraining curvature from within. The approach demands intricate internal locking hardware distributed along the entire robot, these additional components also adding complexity to control.

Recent efforts exploring non actuator based steering mechanisms that rely on either material anisotropy or field-based control, rather than centralized tendon or pneumatic actuation. Mendoza et al. (2024) presented a vine robot capable of achieving high curvature and force through asymmetric elongation of its body, leveraging anisotropic elastic films and layer-jamming. However, the reliance on complex material fabrication restricts scalability and practical miniaturization. Davy et al. (2024) proposed magnetically-actuated steering by embedding magnetic particles within the robot’s skin, offering non-contact control suitable for surgical applications. Nevertheless, external magnetic field requirements restrict deployment scenarios and introduce challenges in environments sensitive to electromagnetic interference.

In this study, we propose an Integrated Bladder and Pivot Joint steering mechanism to control the growth direction through local bending. A tendon-driven pivot joint, attached to the inner inverted material, can bend the robot body at the location of the joint when actuated; inside the pivot joint, there is a pressure-tunable bladder that provides a friction-based connection between the pivot joint and the inner inverted material. Zero bladder pressure (no friction bonding) allows the pivot joint to remain stationary relative to the everting inner body, preserving the bending angle while the robot continues to grow forward. A high-friction bonding (high bladder pressure) would enable the pivot joint to move along with the inverted material during growth until the joint reaches a desired bending location inside the robot body. Our tendon-driven pivot joint, stabilized by a friction-based bladder, delivers reversible and localized steering without embedded motors or distributed actuators. As the two tendons and single bladder are actuated from the base, the steering unit remains lightweight, electronics-free, and leaves the central inverted membrane available as a tool channel. Furthermore, since the steering unit is not permanently affixed to the robot body, the overall growth length is not constrained by pre-distributed actuation, offering clear advantages in terms of scalability. The simplicity of the pneumatic–tendon architecture also makes it inherently amenable for miniaturization due to its simple topology, and for deployment in electromagnetically or spatially constrained environments.

The primary contributions of this study include.

• The design of the novel steering mechanism and its implementation on an SGR prototype. The mechanism is compact in design and can selectively vary the local steering location along the robot body without having to be attached all along the robot body like the SPAM and layer-jamming approaches.
•The tendon-actuated pivot joint is automatically carried by the everted body, enabling steer-lock-release cycles without external intervention.
•The modeling and testing on the inner friction, kinematics, and environment interaction forces of the robot.
•The demonstration of the robot inside a branched arena, demonstrating the effectiveness of the steering mechanism.

## 2 Materials and methods

**FIGURE 1:**
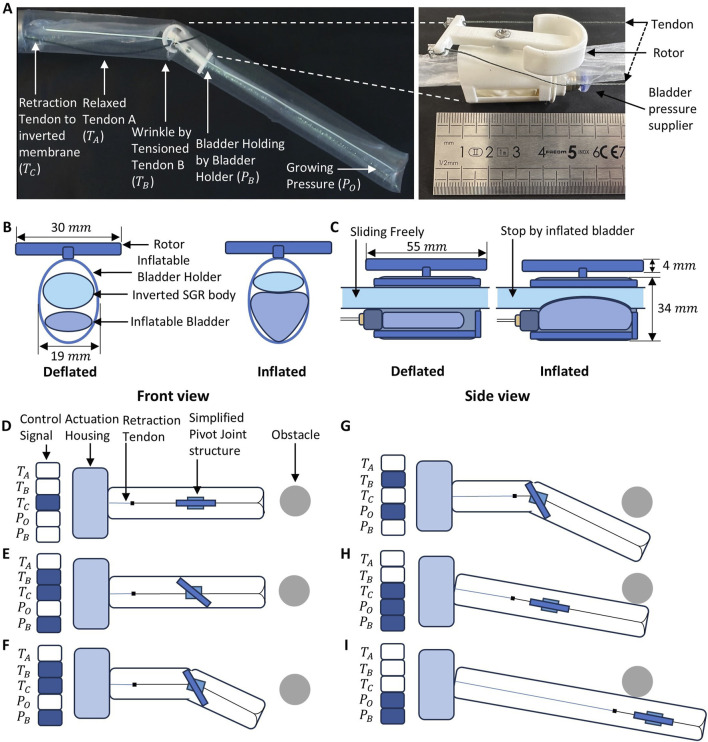
Detailed operational stages and structural components of the pivot-joint integrated soft growing robot: **(A)** Real image depicting pivot-joint and tendon-driven wrinkle; **(B,C)** Front and side views illustrating bladder inflation for friction-based pivot joint anchoring mechanism; **(D)** Initial linear eversion; **(E,F)** Initiation and execution of steering; **(G)** Obstacle negotiation through controlled deformation; **(H)** Post-steering linear growth along new trajectory; **(I)** Shape retention and locking by internal bladder inflation.

The proposed mechanism utilizes an embedded, tendon-driven pivot joint and an pressure-tunable bladder to manipulate the orientation and growth direction of the SGR. Figure 1 illustrates the detailed structural components and operational sequences across several distinctive stages, showcasing the decoupling of axial pivot-joint position from the robot’s eversion and steering.

### 2.1 Mechanism design

The SGR body is essentially an inverted membrane tubing. Releasing the inner inverted membrane material via a central tendon (tendon C) and increasing the air pressure of the SGR would enable the robot to grow at the tip. Inside the SGR body, there is a pivot joint mechanism which has an embedded, pressure-tunable bladder. The pivot joint mechanism is composed of a bladder holder and a rotor driven by two actuating tendons tendon A and tendon B (Figures 1A,B). The rotor is stacked on top of the bladder holder, and they are connected via a rotation shaft, allowing the rotor to freely rotate around the shaft. The rotor is of a boat-anchor shape with two actuating tendons attached on its top. These tendons can control the rotation of the rotor with respect to the bladder holder, and thereby, engage with the robot body’s outer membrane to bend the robot body locally (Figure 1A). The bladder holder is a hollow tube embedded with a pressure-tunable bladder, and the inner inverted membrane material of the robot body goes through the bladder holder as well (Figures 1B,C). As such, inflating or deflating the bladder can change the friction between the bladder holder and the inner membrane material, enabling them to move together (coupled, when the bladder is inflated) or separately (decoupled, when deflated).

### 2.2 Operational stages and steering principle

The detailed steering process depicted in Figures 1D–I is systematically described as follows:

Stage 1: Initial State (Figure 1D): The robot initiates linear eversion with pneumatic pressure $P_{o}$ . At this stage, tendons A , B remain relaxed, retraction tendon C slightly tensioned to prevent excessive robot eversion.

Stage 2: Steering (Figures 1E,F): When encountering an obstacle, to avoid and passing through, the bladder pressure $\left(P_{B}\right)$ is increased, enhancing friction bond to securely anchor the pivot joint mechanism with the inner inverted material of the robot; this ensures that the pivot joint does not translate under the tendon tension during the bending of the robot. Then, one steering tendon, e.g., tendon B , is tensioned to firmly engage the pivot mechanism against the robot’s internal membrane with the rotor and to bend the robot body at the location of the pivot joint.

Stage 3: Decoupled Growth for Obstacle Avoidance (Figure 1G): The robot grows by increasing pneumatic pressure $P_{O}$ and releasing the retraction tendon C . The bladder is deflated so that pivot joint does not move forward together with the inner membrane material (decoupled); in the meantime, the pivot joint keeps stationary because the rotor is anchored to the membrane wall of the robot body, which maintains the bending of the robot so that it grows to the steered direction to avoid the obstacle.

Stage 4: Coupled Growth (Figures 1H,I): After successfully passing the obstacle, all tendon tensions are released. The bladder is re-inflated to restore frictional coupling between the pivot joint and the inner membrane. Pressure $P_{O}$ is increased to resume the pivot joint’s forward movement together with the everting body along the newly established trajectory. The robot body then relies on the surrounding environment to preserve the bending configuration, allowing it to continue growth or repeat the steering cycle as needed.

### 2.3 Mechanics modelling

#### 2.3.1 Tensioned tendon for steering

**FIGURE 2:**
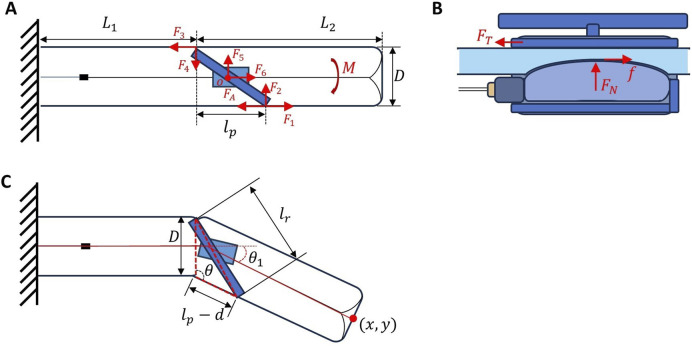
Mechanics analysis diagram illustrating wrinkling forces, frictional engagement, and resultant bending moments influencing robot steering: **(A)** Simplified free-body diagram of forces acting on the pivot joint; **(B)** Side-view of frictional locking mechanism via bladder inflation; **(C)** Geometric definitions interactions associated with steering.

To evaluate the bending moment applied by the rotor to the robot body, we first isolate the rotor structure as a free body, as illustrated schematically in Figure 2A.

$T_{A}$ represents the pulling force of tendon A to the rotor; as such, the rotor is rotated to engage with the robot’s outer membrane and is simultaneously braced by four reaction forces transmitted through the outer membrane, including two axial components $F_{1},F_{3}$ acting tangentially along the membrane and the radial components $F_{2},F_{4}$ acting normal to the membrane. Each axial-radial pair forms a force couple, and they together generate an anticlockwise moment to the rotor, i.e., $M_{s}$ .

In addition, the rotor is free to rotate around a rotation shaft connected to the bladder holder. The rotation shaft applies an orthogonal pair of forces to the rotor $F_{5},F_{6}$ .

The rotor has movement-equilibrium under these forces. Take point O as the moment axis, we have Equations 1, 2

$$\sum M_{O}=0\;\Rightarrow M_{s}-T_{A}\cdot r=0$$ (1)

Thus,

$$M_{s}=T_{A}\cdot r$$ (2)

Note that $M_{s}$ is the resultant moment of the forces from the membrane to the rotor; thus, the membrane also bears the same moment from the rotor in the opposite direction (clockwise when tendon A is pulled, magnitude is $T_{A}\cdot r$ ). Such a moment bends the robot body to the clockwise direction (when tendon A is pulled).

#### 2.3.2 Wrinkling force for steering

The steering of the SGR with pivot joint fundamentally relies on forming localized wrinkles via tendon-driven actuation, combined with internal pneumatic pressure, the result also flows the equation proposed by Wang et al. (2020).

As depicted in Figures 2A–C, the robot’s body deformation arises from the interaction of internal pneumatic pressure $\left(P_{o}\right)$ , tendon-induced tension (T) , and inherent material characteristics. The required tension T to create and maintain a wrinkle in the robot body can be mathematically represented as follows:

$$T=\frac{\pi D^{2}P_{o}}{8}+K$$ (3)

Where D is the cross-sectional diameter of the robot. The parameter K represents a material-dependent offset factor arising from the anisotropic properties of the robot’s material. Notably, K is considered independent of the internal pressure and remains approximately constant across operational conditions.

The implication of Equation 3 is that, at a given internal pressure $P_{o}$ , a consistent, minimal tendon tension T is sufficient to maintain the wrinkle. This also indicates that the required tension is independent of the bending angle, i.e., the tension does not change with respect to the steering angle.

#### 2.3.3 Inner bladder friction analysis

Throughout the operational stages (Figures 1D–I), the robot’s main pneumatic pressure $\left(P_{o}\right)$ induces tensile stress along the membrane and everts the inner material out for forward growth $\left(F_{P}\right)$ . Tendon tension (e.g., $T_{A}$ ) drives the rotor to compress the outer membrane to form a wrinkle $\left(F_{1},F_{2},F_{3},F_{4}\right)$ for bending. Conversely, alternative tendon tensions (e.g., $T_{B}$ ) can reverse the robot’s curvature.

During the above-mentioned Stage 2 (steering), the internal inflatable bladder is inflated with a high pressure $\left(P_{B}\right)$ generates a significant radial normal force $\left(F_{N}\right)$ , leading to a friction bonding between the bladder and the inner membrane material, thereby fixing the pivot joint on the inner membrane to facilitate robot body bending on the spot. In this case, the maximum friction force generated by the bladder pressure should be sufficient to resist the tendon pulling force to prevent the pivot joint being pulled backowards the base, i.e. Equation 4

$$f=\mu F_{N}=\mu P_{b}A_{b}\geq T$$ (4)

where μ is the friction coefficient between the inflated bladder and the inverted SGR’s internal membrane, and $F_{N}=P_{b}\times A_{b}$ represents the normal force between the balloon and the inner membrane material due to the bladder air pressure ( $A_{b}$ is the contact area of the bladder with the inner membrane material). Note that such friction is also needed in Stage 4 where the pivot joint is supposed to move forward together with the inner membrane material.

During Stage 3 (decoupled growth), the bladder is deflated (Figure 2B) so that the normal force $F_{N}$ is negligible, resulting in minimal friction f≈0 . In this case, the pivot joint and the inner membrane material are decoupled. Releasing the central tendon would enable the inner material to evert out for tip growth while the pivot joint is kept stationary due to the steering tendon’s tension and the rotor’s engagement with the robot’s outer membrane. At this stage, the base tether tension $F_{C}$ is relaxed $\left(F_{C}=0\right)$ , and the forward motion is driven solely by the internal pneumatic force $F_{P}$ , expressed as Equation 5:

$$F_{P}=P_{o}\cdot A=\frac{\pi D^{2}P_{o}}{4}$$ (5)

### 2.4 Kinematics modelling

To capture the characteristic of the soft growing robot equipped with a pivot joint mechanism, a kinematic model was theoretically derived. The model maps the actuation space (tendon displacement) to the configuration space (robot spinning angle and pivot joint position) and ultimately to the task space (tip poses). The model was then experimentally verified.

As shown in the robot schematic diagram (Figure 2), the robot can be treated as two sections connected by a pivot joint. The pivot joint is controlled by one tendon on each side, with its position coupled with the releasing of the central retraction tendon when the bladder is inflated during steering stage. The total length of the robot L is $L_{1}+L_{2}$ , where $L_{1}$ is the length of the first section and $L_{2}$ represents the second. The growth of the whole robot is controlled by the release of the central retraction tendon $L=d_{c}/2$ , where $d_{c}$ is the release length of the central retraction tendon.

To bend the robot body, the rotor must first contact with the body inner membrane (Figure 2A). Further pulling the tendon would bend the robot. Here, we built the relationship between the tendon input displacement and the robot bending angle based on the geometry of a triangle (see the dashed red lines in Figure 2C) formed by the width of the robot (SGR diameter) D , the rotor contact length $\left(l_{r}\right)$ , and the projection length $l_{p}$ of rotor along the bended section of the robot. Pulling the tendon would shorten $l_{p}$ and thus increase θ . Here, the robot bending angle is $\theta_{1}=\theta -90{}^{\circ}$ . For this triangle, we have Equation 6

$$l_{r}^{2}=D^{2}+{\left(l_{p}-d\right)}^{2}-2D\left(l_{p}-d\right)\cos\theta$$ (6)

We can get the angle of the pivot joint with the robot outer membrane (Equation 7):

$$\theta =\arccos\left(\frac{{\left(l_{p}-d\right)}^{2}+D^{2}-l_{r}^{2}}{2\left(l_{p}-d\right)D}\right)$$ (7)

As the rotor contact the membrane, the initial $l_{p}$ (Figure 2A) can be define as Equation 8:

$$l_{p}=\sqrt{l_{r}^{2}-D^{2}}$$ (8)

The pivot joint can produce a controllable bend of up to 90° . Further tendon pulling displaces the pivot away from the wrinkle, after which the tube buckles rather than steers; therefore, curvature beyond 90° is not controllable and was excluded from our operational envelope.

As the robot steering angle $\theta_{1}=\theta -90{}^{\circ}$ , the end effector position can be determined. By giving the position of the pivot joint, the whole robot can be simplified as an two link robot, and the tip point can be calculated as Equation 9:

$$\left\{\begin{array}{c} x=L_{1}+L_{2}\cos\left(\theta_{1}\right)\;\\ y=L_{2}\sin\left(\theta_{1}\right).\; \end{array}\right.$$ (9)

### 2.5 Experiment setups

#### 2.5.1 Kinematics verification and critical buckling force evaluation

**FIGURE 3:**
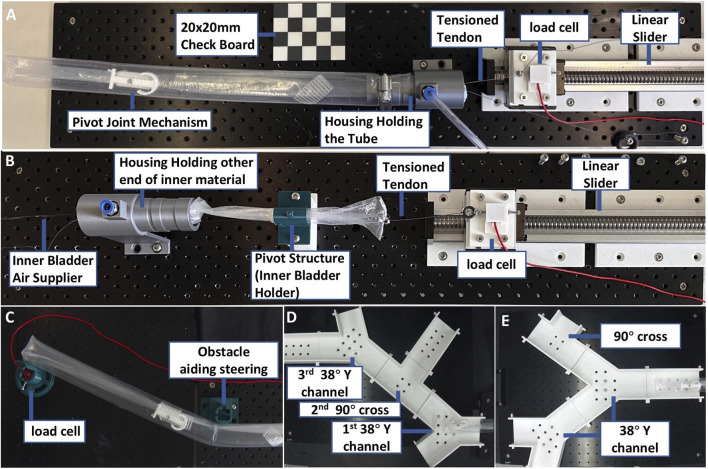
Experimental setups for evaluating robot performance. **(A)** Setup for kinematics and buckling tests using tendon actuation and visual tracking. **(B)** Frictional anchoring test to evaluate bladder-induced holding force on the pivot joint. **(C)** Setup for measuring contact forces during environmental interaction. **(D,E)** Different maze-like navigation arena settings for steering performance demonstration in unstructured environments.

As shown in Figure 3A, a 445 mm by 45 mm plastic robot body with a 55 mm by 30 mm pivot joint printed by Bambu X1 with PLA filament, robot was mounted onto a custom-designed housing, where the steering tendon passed through a guide channel and was connected to a load cell (Futex LSB201) attached to a linear actuator. A top-down camera was used to track the shape profile of the robot with a checkerboard for coordinate calibration. This setup enabled real-time measurement of both tendon displacement, tendon tension, and robot shape. Matlab Simulink was used to synchronise all data acquisition processes, including tendon force, displacement, and robot tip angle. For the kinematics verification, the tendon was incrementally pulled for 30 steps, and each step’s displacement was 0.54 mm ; then, the tendon was incrementally released in 30 steps, and each step’s displacement was also 0.54 mm . The recorded video was analyzed using sort-line tracking to extract the shape of the robot’s body during the action. A red paper line was attached to the plastic robot body central and then skeletonized to represent the centerline of the robot body. The coordinates are mapped and calculated based on checkerboard-based projection. The 30 steps of the extracted motion trajectory was subsequently compared with the predicted trajectory derived from simulations under identical displacement inputs steps. For the buckling force measurement, the buckling force was tested by continuously pulling the tendon for 23.1 mm on different pressures varying from 0 to 0.7 psi) .

#### 2.5.2 Bladder friction test

To evaluate the friction force that can be generated by the bladder at different pressures (0–1 psi at an interval of 0.1 psi ), the experimental setup shown in Figure 3B was used. A piece of membrane material was placed in-between the bladder made with nitrile rubber and the bladder holder. The membrane was then pulled by a linear motor on one side to slide with respect to the bladder. The displacement and force were recorded. The housing on the other end of the inverted material is to hold maintaining as the same level of the load cell to avoid errors. The membrane material was pulled for 40 mm to simulate the growing procedure. The resulting pulling force was recorded and considered as the effective friction force provided by the bladder to hold the pivot joint in place.

#### 2.5.3 Evaluation of environmental contact forces for postural support

During the above-mentioned Stage 4, The robot body relies on the surrounding environment to preserve the bending configuration. It is important to evaluate the interaction forces between the environment and the robot. In this experiment, a loadcell was employed to measure the required external physical support of the obstacle to passively maintain the robot’s posture during growth (Figure 3C). The robot was tested under various internal pressures ranging from 0.4 psi to 1.0 psi , simulating different stages of growth and stabilization. The interaction forces were measured at two locations: the tip of the robot and the midpoint of the steered segment. Two different bending angles were considered: 17° and 48°. The total length of the robot beam was 665 mm and the steering point was at location 330 mm from the base.

#### 2.5.4 Exploration and navigation in unstructured environments

To evaluate the robot’s ability to explore unfamiliar and dynamically arranged surroundings through real-time control and navigation, modular reconfigurable mazes (Figures 3D,E) were constructed using interlocking components to emulate an unstructured environment. Several 3D-printed modular components, including Y-shaped junctions (each providing a 38° bend angle), T-shaped junctions, and straight segments, were used to assemble the experimental mazes. These modular components were rearranged to create the two distinct mazes configurations presented in Figures 3D,E.

## 3 Results and discussion

### 3.1 Kinematics verification

**FIGURE 4:**
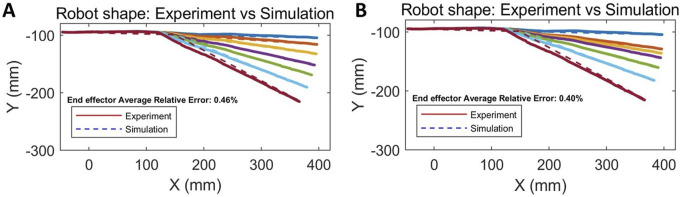
Kinematic verification through experiment and simulation. **(A)** Bending process. **(B)** Unbending process. Experimental results are compared with simulation to evaluate model accuracy.

The kinematic model was validated by comparing its simulations against experimental deformation profiles under both bending and unbending conditions. The predicted robot end effector position (Equation 9) were compared against experimentally recorded deformation profiles under both bending (Figure 4A) and unbending motions (Figure 4B). As shown in Figure 4, the simulated end effector position closely matched the experimental observations, with average relative errors of 0.46% during bending and 0.40% during unbending, respectively. The relative error for each end effector is calculated as the average distance from the position of the end effector in simulation to the corresponding end effector in the experiment, divided by the robot length. These results confirm that the proposed model accurately captures the bending configuration of the robot and provides a reliable basis for motion planning and control.

### 3.2 Effects of air pressure to steering

**FIGURE 5:**
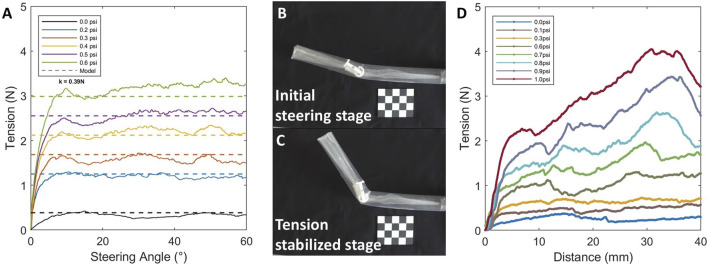
Evaluation of pressure effects on robot steering performance. **(A)** Effect of internal pressure on the critical tension required for buckling. **(B)** Tendon tension increasing stage with initial steering **(C)** Tendon tension stabilized stage with increasing steering angle **(D)** Effect of bladder pressure on the frictional force for pivot joint anchoring.

The robot exhibited a clear buckling behavior in response to tendon actuation. In the initial stage from 0° to approximately 8° (Figure 5B), the required tension increased rapidly with the bending angle. Once the bending exceeded this critical buckling angle (Figure 5C), the additional tension needed for further bending tended to stabilize (Figure 5A). This critical point corresponds to the condition where the internal compressive force offsets the axial tensile stress, leading to the onset of local wrinkling. The critical tension required to initiate buckling increased with pressure, following the relationship defined in Equation 3. To experimentally determine the parameter K , we repeated the bending tests without internal pressure (i.e., P=0psi ). During these tests, we measured the bending forces exerted by the tendon to form and maintain the wrinkles. The results indicated a consistent tendon force value of approximately 0.39 N , which represents the inherent stiffness of the pivot joint structure in the absence of internal pressurization. Based on these new measurements, the parameter K was set to 0.39 N and subsequently utilized for the data fitting presented through Equation 3. Once buckling occurred, the tension required to further enlarge the deformation remained nearly unchanged. In this work, we choose a low air pressure (0.3 psi ) during steering to keep required steering force low.

In addition to the internal pressure used for tip eversion and growth, the bladder pressure, which controls the anchoring of the pivot joint, also plays a critical role in the robot’s functionality. As previously described, higher inflation pressures generate larger frictional forces between the bladder and the inverted inner tube membrane, effectively securing the pivot joint to the robot body and enabling co-growth. Conversely, reducing the bladder pressure releases internal constraints, allowing the robot to extend independently of the joint.

The test measured the axial frictional resistance provided by the bladder–tube interface at different pressure levels (Figure 5D). Results showed that when the bladder pressure reached 1 psi , the resulting frictional force exceeded 2 N , which was sufficient to reliably anchor the pivot joint during growth. These findings provide a practical guideline for selecting actuation pressures that ensure sufficient friction to anchor the pivot joint during co-growth, while still allowing for its controlled release when further extension is required.

### 3.3 Role of environmental constraints in steering and growth

The robot can actively steer and grow via tip eversion. However, after bending, it lacks dedicated mechanisms to hold its shape. Instead, posture is passively maintained through contact with the environment—a form of morphology stabilization enabled by external constraints.

**FIGURE 6:**
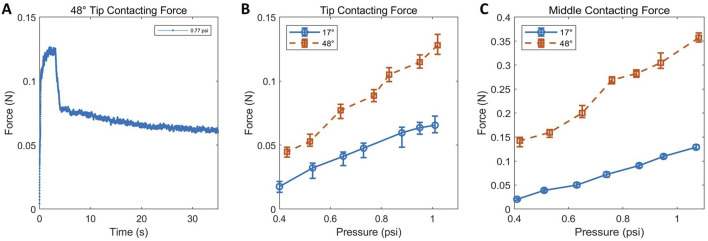
Contact force evaluation during robot-environment interaction. **(A)** Full force profile during contact. **(B,C)** Steady-state contact forces under different bending angles and contact positions.

To quantify the role of environmental contact in maintaining robot posture, we measured the interaction force between the robot body and external supports under different internal pressures and contact positions, corresponding to different steering angles. As shown in Figure 6A, the contact force exhibited a transient peak upon initial contact, with values not exceeding 0.15 N , followed by a steady-state phase where the force stabilized around 0.06 N . Furthermore, the measured contact force decreased with increasing distance from the pivot point (Figures 6B,C),in agreement with the mechanical principle that torque is proportional to force times distance. The required contact force also increased with larger bending angles, indicating that greater deformation demands higher external force to maintain the resulting posture. These findings indicate that environment-assisted stabilization relies on relatively low contact forces, sufficient to retain the robot’s posture without exerting excessive mechanical load on its surroundings.

Overall, while the robot relies on passive environmental constraints to maintain its postural configuration after deformation, the contact force involved remains low. This suggests that the stabilization mechanism does not significantly burden or damage the environment, nor does it require strong or specially structured external supports. The low-contact nature of this stabilization strategy enhances the system’s adaptability and ensures compatibility with a wide range of operating conditions without requiring structural modifications to the environment.

### 3.4 Demonstration of pivot joints navigation capabilities

**FIGURE 7:**
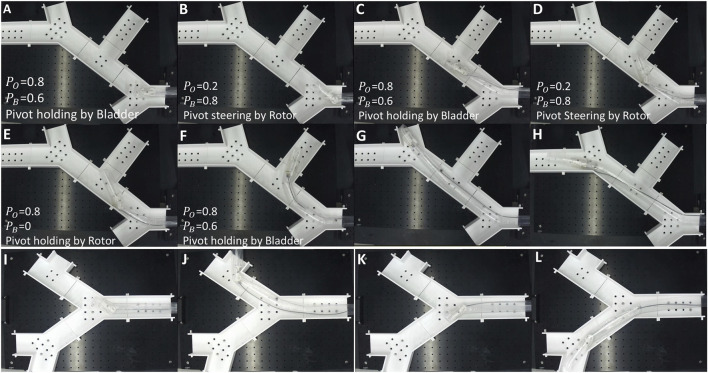
Demonstration of robot navigation using pivot joints in unstructured environments. **(A,B)** The robot successfully steers through the first corner of a maze. **(C)** Continued growth after initial steering. **(D–F)** Secondary bending enables navigation toward a bifurcated dead-end. **(G,H)** The robot explores the alternative paths in the same maze under manual steering. **(I–L)** Navigation in a different maze structure, demonstrating robustness across environments.

The navigation capabilities of the soft growing robot equipped with pivot joints were evaluated in structured maze-like environments. As shown in Figures 7A,B, the robot performed an initial turning rotor to cross the first corner of a maze. Figure 7C demonstrates its ability to continue tip eversion and growth after steering. Subsequently, in Figures 7D–F, the robot executed a secondary bending motion, successfully navigating toward the end of a bifurcated path.

These sequential deformations highlight the robot’s capacity for complex navigation and repeated directional change. In Figures 7G,H, under manual control, the robot was steered to explore an alternative branch within the same maze, demonstrating its capability for operator-guided decision-making in unstructured settings. Finally, Figures 7I–L ppresents the robot operating within an altered maze configuration constructed from the same modular components. In this scenario, the robot continues to exhibit consistent exploratory behavior, with Figures 7I,J depicting the robot choosing the upper branch at the first Y-junction and subsequently executing another 90° turn in the same direction. Conversely, Figures 7K,L demonstrates the robot initially choosing the alternate path at the first Y-junction and performing a further bend along this route. The repositioning of panels was intentionally chosen to clearly illustrate the robot’s adaptability across different environmental layouts.

During steering, the pivot joint rotor must first establish firm contact with the robot’s inner wall before tendon displacement effectively induces bending of the leading segment. This required sequence inherently introduces some delay, which may slightly impact real-time manual control responsiveness.

After repeated testing, we observed that multiple bending and inflation cycles could potentially cause material degradation in the robot’s root membrane, increasing susceptibility to unintended bending. Additionally, repeated bladder inflation-deflation cycles occasionally caused partial extrusion of the bladder from its holder, potentially leading to punctures and subsequent air leakage. Such damage could impair the reliability of the locking mechanism.

These results confirm that the robot’s steering and growth are not limited to preprogrammed trajectories. Instead, the system can be manually controlled in real time to explore unfamiliar, unstructured environments, reflecting strong potential for use in navigation tasks where environmental layouts are unknown or dynamic.

## 4 Conclusion

In this study, we presented a novel steering mechanism for SGRs. The proposed design integrates a tendon-driven pivot joint with a pressure-tunable internal bladder, leveraging frictional engagement to selectively couple or decouple the joint from the everting body. This enables smooth transitions between local bending and forward growth with a compact unit, in contrast to body-long steering mechanisms that require distributed actuators along the entire body to achieve shape change.

Through analytical modelling and experimental validation, we demonstrated that the pivot joint can reliably generate controlled steering with low tendon tension and subsequently grow to the steer direction. The proposed kinematic model accurately predicted robot configuration with less than 0.5% error, and the robot successfully navigated maze-like, cluttered environments with multiple steering events. Frictional analysis further confirmed that bladder inflation pressures above 1 psi provide sufficient anchoring force (>2N) to secure the joint during actuation, while environmental contact forces required for postural stabilization remained minimal (>0.1N) , confirming the system’s gentle interaction with surroundings.

For the SGR with pivot joint steering mechanism, it relies on environmental contact to passively retain its posture after bending. As the pressurized body generates a self-straightening moment that tends to restore the structure to its original configuration during continued growth. Additionally, the use of a bladder to compress the inverted inner membrane of the tip-everting SGR may interfere with the future integration of tool channels extending from the base, limiting internal payload deployment.

Future work will focus on expanding the system’s directional control into three dimensions using pivot actuation with torque coil attached on the end of the pivot joint structure. By imposing controlled axial rotation, the coil will realign the mechanism and allowing navigation in 3-D space. Moreover, tendon would also be enclosed to reduce direct contact with the robot’s outer membrane, thereby reducing friction accumulation.

## Data Availability

The original contributions presented in the study are included in the article/supplementary material, further inquiries can be directed to the corresponding author.
